# Contribution of ambient noise and hyperbaric atmosphere to olfactory and gustatory function

**DOI:** 10.1371/journal.pone.0240537

**Published:** 2020-10-13

**Authors:** Hans-Georg Fischer, Christopher Schmidtbauer, Annett Seiffart, Michael Bucher, Stefan K. Plontke, Torsten Rahne

**Affiliations:** 1 Department of Otorhinolaryngology, Head and Neck Surgery, University Hospital Halle (Saale), Martin Luther University Halle-Wittenberg, Halle (Saale), Germany; 2 Department of Otorhinolaryngology (ENT), Head and Neck Surgery, Military Hospital Hamburg, Hamburg, Germany; 3 University Clinic for Anesthesiology and Operative Intensive Care, University Hospital Halle (Saale), Martin Luther University Halle-Wittenberg, Halle (Saale), Germany; Monell Chemical Senses Center, UNITED STATES

## Abstract

**Introduction:**

Taste and smell are important for occupational performance and quality of life. Previous studies suggested that the function of these senses might be influenced by ambient pressure and noise. This knowledge would be helpful for divers, submarine crews, or mine workers. The present study aimed to investigate the effects of noise and hyperbaric pressure on olfactory and gustatory functions.

**Methods:**

This prospective controlled study included 16 healthy male divers. Inside a hyperbaric chamber, participants performed olfactory and gustatory function tests at sea level pressure and at 2 bar pressure. The olfaction threshold, and the discrimination and identification of odorants were measured with validated ´Sniffin sticks´. Taste identification and the gustation threshold scores were examined with validated filter paper strips. Tests were performed under two conditions: noise reduction (silence) and white noise stimulation presented at 70 dB sound pressure level.

**Results:**

The results showed that normobaric and hyperbaric ambient pressures did not significantly affect olfactory or gustatory function. Moreover, noise had no relevant impact on taste or odor sensation. The odor identification score was not influenced in hyperbaric conditions, and the odor threshold score was not influenced by ambient noise or both barometric conditions. The only taste modality affected by hyperbaric conditions was the sensitivity to salty taste, but it was not significant.

**Conclusion:**

We concluded that hyperbaric and noisy environments have no influence on gustatory and olfactory function. From a practical point of view, the influence of pressure in moderate hyperbaric occupations should be negligible.

## Introduction

Olfaction plays an important role in human daily life. For example, nutrition, safety, and interpersonal relations are influenced by aversions and attractions to odorous items [1]. Previous studies have discussed and partially demonstrated various environmental influences on human olfactory and gustatory functions. Temperature, humidity, barometric pressure, and air currents influence the movements of molecules and their processing by the perceiver [2]. For instance, a low humidity environment significantly reduced the odor threshold compared to a humid environment [3]. Furthermore, environmental pollution was shown to influence odor detection, discrimination, and identification [4, 5]. Ambient pressure might also potentially play a role in odor or taste perception. For example, hypobaric pressure was shown to have a negative influence on odor thresholds [6].

Normal olfaction is required for good job performance and occupational safety in certain professions, such as mining, submarine operation, firefighting, and underwater saturation diving. Therefore, the influence of hyperbaric conditions on olfaction should be explored. Positive environmental effects on olfactory perception have rarely been explored [7], particularly hyperbaric effects on olfactory and gustatory functions.

To our knowledge, only two peer-reviewed studies previously investigated the effects of hyperbaric pressure on odor thresholds and discrimination tasks [3, 8]. The results of those studies suggested that odor threshold scores were increased under hyperbaric conditions. Initial studies on gustatory function in hyperbaric conditions were conducted in 1977 by Halbreich et al. [9] and O’Reilly et al. [10]. Halbreich et al. found that hyperbaric conditions increased the thresholds for all taste modalities. In contrast, O’Reilly et al. found that hyperbaric conditions increased the sensitivity to bitter stimuli. This discrepancy was suspected to be due to confounding effects, like stress.

According to a survey conducted on saturation divers, in the saturated state 46% perceived distortions in the taste and 42% in smell of foods. Interestingly, those who perceived distorted tastes and smells mentioned that they experienced reduced taste and smell sensitivities [11]. Certainly, those results could be affected by multiple factors, but cause and effect could not be ascertained in that investigation.

In addition to ambient pressure, other environmental factors that might influence taste and smell have been discussed. The concept of multisensory stimulation was introduced as an alternative explanation for different influences on the senses, including suppressive and super-additive influences. According to this concept, the sense of flavor can be modulated by gustatory (taste), olfactory (smell), and sometimes oral-somatosensory (touch) inputs. Based on psychological and neuronal influences, several studies on different senses have revealed that some stimuli can have profound effects on the perception of multiple sensory modalities [12–14].

Spence (2015) reviewed a complex multisensory interaction related to flavor experiences, which included visual, trigeminal, and auditory contributions [15]. In pressure chambers and cabins, loud noises are common, and they could potentially cause the described smell and taste distortions. Velasco et al. (2014) showed that white noise had a more pronounced effect on participant odor ratings than consonant or dissonant musical selections [16]. Seo et al. (2012) found evidence that odor sensitivity could be modulated by different types of background noise (nonverbal vs. verbal noise) and different degrees of extraversion [17]. Rahne et al. (2018) concluded that hypobaric or noisy environments could selectively impair gustatory and olfactory sensitivity for particular tastants and odorants [18]. That study demonstrated small, but significant reductions in gustatory and olfactory sensitivities in a hypobaric atmosphere compared to normal pressure. They also reported that white noise did not influence the odor test results, but impaired the sensitivity to sour and sweet tastants.

We hypothesized that a hyperbaric atmosphere might increase olfactory and gustatory sensitivity, and ambient noise might reduce olfactory and gustatory sensitivities for particular tastants. The present study aimed to determine the effects of noise and hyperbaric atmosphere pressure on olfactory and gustatory functions in a well-defined, controlled experimental setting.

## Materials and methods

### Participants

This prospective, single-blinded, clinical study included 16 male volunteers, aged 22 to 35 years (mean, 26.4; SD 4.4 years), in good health, with normal hearing, and no olfactory or gustatory disorders in medical history. Assuming an olfactory threshold, discrimination, and identification (TDI) score difference of 5 with standard deviations of 7 as clinical relevant [19], a mean effect size of d = 0.5 was assumed for sample size estimation. Assuming an α = 0.05, and a power of 0.8 (β = 0.2), we estimated that a sample size of 16 was sufficient to measure a TDI difference of 5 if the standard deviation was 5 in two indipendant groups (G*Power Software Version 3.1, Heinrich-Heine-University, Düsseldorf, Germany). To reduce the risk of health damage due to hyperbaric pressure, this study only included divers that had performed at least two dives to more than ten meters below sea level in the last 12 months. All subjects provided written consent after receiving information about study procedures and aims. Subjects had study-related insurance and were paid for participation. The study was approved by the Ethics Committee of the Martin-Luther-University Halle-Wittenberg (approval number 2017–121). It was conducted in accordance with the Declaration of Helsinki.

All participants underwent a preliminary medical examination to exclude cardio-vascular and pulmonary diseases. An ear, nose, and throat examination was performed by an ENT specialist. Middle ear pressure equalization was assessed by observing the tympanic membrane during a Valsalva maneuver with an ear microscope. Furthermore, tympanometry was performed to exclude individuals with middle ear effusion or tympanic membrane retraction to lower the risk of ear squezze. All participants underwent pure-tone audiometry to ensure normal hearing; i.e., air conduction hearing thresholds were below 25 dB at 0.5, 1, 2, and 4 kHz. A nose inspection and endoscopic examination of the nasal cavity were performed to exclude pathologies of the inner nose, like a septum deviation or signs of rhinosinusitis, which could negatively affect nasal function. Additionally, the oral cavity, including the tongue and oropharynx, were inspected to rule out, e.g., oral thrush or glossitis as a potential risk for gustatory impairments. Further exclusion criteria were: active smoking, claustrophobia, odor or tastant allergies, and current use of streptomycin, D-penicillamine, diltiazem, nifedipine, amitriptyline, methotrexate, amphetamines, alcohol, local vasoconstrictive substances, strychnine, codeine, or lidocaine.

### Experimental setup

The experiments imposed pressure and noise conditions on participants inside a hyperbaric chamber with a volume of 22.15 m^3^ (HBO 1, SAYERS/HEBOLD, Germany) at the University Hospital Halle (Saale), Department of Diving and Overpressure Medicine. During all measurements, an air conditioner was operating at maximum air exchange (840 l/min) to maintain a stable odor status in the cabin. Between experiments in hyperbaric and normal conditions, a complete air exchange was performed as well as a break of 20 minutes to acclimatize the participants. For all measurements, pressure, temperature, humidity, and oxygen concentration were monitored and recorded. A qualified diving medicine physician supervised all participants during pressurization.

### Pressure environment

Ambient pressure was maintained at 2 bars for the hyperbaric pressure condition and at 1 bar for the normobaric condition. According to the decompression table [20], for this pressure and duration, no decompression stops were necessary. These particular pressure levels were chosen, based on laboratory experience, because subjects tended to gradually develop signs of cognitive dysfunction above 1 bar of pressure. After reaching ambient pressure, the experiments were started immediately.

### Noise environment

White noise was generated with a MATLAB program, at a sound pressure level of 70 dB. In noise conditions, participants were continuously exposed to white noise by wearing E-A-RTONE 3A-insert earphones (3M, St. Paul, MN, USA). The audio interface was adjusted with an artificial ear simulator to a sound pressure level of 70 dB. In silence conditions, participants were protected from cabin noise by wearing E-A-R earplugs (3M, St. Paul, MN, USA) and SPERIAN circumaural (Howard Leight, Smithfield, RI, USA) hearing protectors.

### Functional measurements

Olfactory and gustatory function measurements were performed in randomized order. Additionally, every combination of noise and pressure conditions and subsets were applied in random order (Table 1). All subjects were blindfolded during testing.

**Table 1:** Experimental setting with associated barometric and acoustic conditions.

Measurement Condition	Ambient Pressure (bar)	Sound Pressure Level (dB)	Exspected Examination Time (min)
Hyperbaric/Silence	2	0	45
Hyperbaric/Noise	2	70	45
Normobaric/Silence	1	0	45
Normobaric/Noise	1	70	45

Olfactory function was evaluated with ´Sniffin Sticks´ (Burghart Messtechnik, Wedel, Germany) [21–23]. In three different experiments, identification, discrimination, and threshold subtests were performed. Odors were presented with odor-dispensing pens, 14 cm long, with an inner diameter of 1.3 cm. Pens were filled with liquid odorants or odorants dissolved in propylene glycol to a total volume of 4 ml. Investigators presented the odors by removing the pen cap and holding the pen under the participant’s nostrils at a distance of 2 cm. The pen was moved in a small circular pattern for about 3 seconds. The investigators wore cotton gloves to avoid contaminating odors.

Olfactory thresholds were measured with 16 triplet samples prepared with different concentrations of n-butanol [24]. For each concentration, the participant was presented with three different pens. For a correct response, the participant had to identify the one pen in the triplet that contained n-butanol. Starting with a high concentration, in each successive test, the concentration was incrementally reduced until a false response was reported. Then, the concentration was incrementally increased, until a correct response was reported. The procedure was repeated six times. The results were analyzed with logistic regression [25] to calculate the olfactory threshold score, which ranged from 1 (highest concentration) to 16 (lowest concentration).

Odor discrimination was measured with a test battery of 16 triplet samples, presented in randomized order [24]. In each trial, one pen of the triplet had a different odor than the other two pens. The total score was the sum of correct answers for each participant, ranging from 0 to 16.

The odor identification task consisted of a multiple-choice test, where 16 odors were presented successively [24]. For each pen presented, the participant had to identify the odor among four possible choices. The identification score was the sum of correct responses for each participant, ranking from 0 to 16.

A composite olfactory threshold, discrimination, and identification (TDI) score was calculated separately for every combination of pressure and noise conditions [24].

Gustatory function was measured with ´Taste strips’ [26], which were filter paper strips impregnated with one of four basic tastants: sweet (sucrose), bitter (quinine hydrochloride), sour (citric acid), and salty (sodium chloride), each prepared at four different concentrations [27]. The test also included two control strips without a tastant. The strips were placed on the participant’s tongue in random order. The participants responded by identifying one of the four flavors or ‘no flavor’. The gustatory score was the sum of correct responses, ranging from 0 to 18 [28].

### Statistics

Statistical data analyses were performed with SPSS 23 software (IBM, Ehningen, Germany). Normal data distributions were tested with the Shapiro-Wilk-Test. Cumulative TDI scores, discrimination scores, and olfactory thresholds were compared between groups with repeated measures ANOVAs. Mean gustatory scores and odor identification scores were compared between groups with the non-parametric Friedman-test. The olfaction groups comprised the same subjects tested under different conditions of pressure (Hyper, Normal) and noise (Noise, Silence). The gustation groups comprised the same subjects tested for different tastes (Sweet, Sour, Salty, Bitter) under different conditions of pressure (Hyper, Normal) and noise (Noise, Silence).

When the Mauchly test showed a significant result, the degrees of freedom were reduced with the Greenhouse-Geisser correction. For post hoc analyses, least significant difference tests were applied. For all comparisons, α was set to 95%.

## Results

All subjects completed the measurements. In the medical examination, the pure-tone audiograms showed that air conduction hearing loss was <25 dB for all participants at frequencies of 125 Hz to 8000 Hz, which indicated normal hearing. The ear, nose, and throat examination revealed no pathological findings. Within the chamber, the mean air temperature was 28.4°C (SD: 1.0°C), the mean humidity was 47.1% (SD: 7.7%), and the mean oxygen concentration was 20.9% (SD: 0.12%). No medical issues (adverse events) occurred during the experiments.

**Fig 1:**
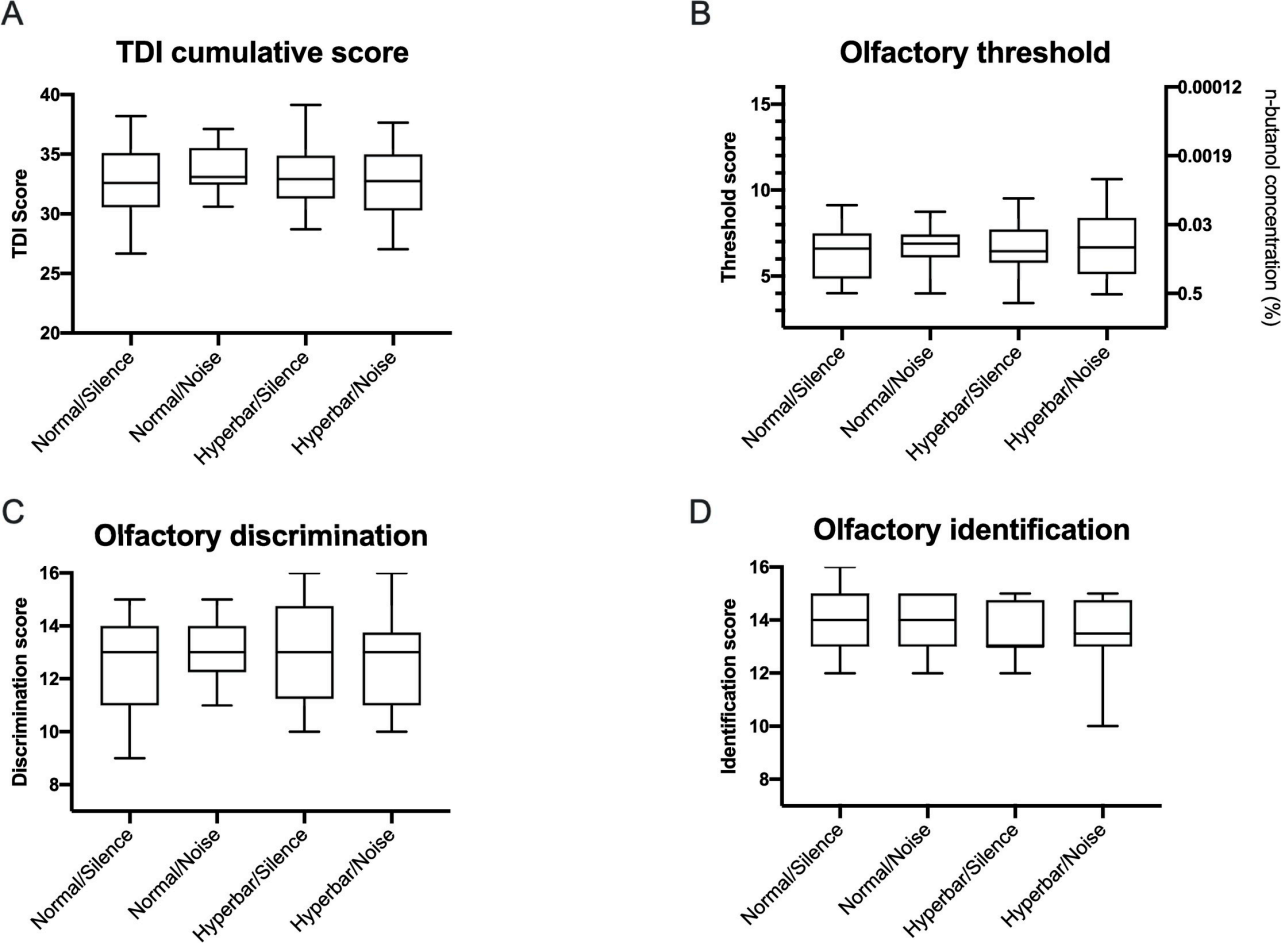
Olfactory function scores based on ´Sniffin Sticks‘ shown as Box-Whisker-Plots. (A) Cumulative TDI, (B) threshold, (C) discrimination, and (D) identification scores. The boxes show the first and third quartile and the median. The whiskers show the minimum and the maximum.

Fig 1 shows the total olfactory TDI score and the underlying subscores for the odor threshold, odor discrimination, and odor identification.

Table 2 shows the mean TDI scores and the gustatory threshold scores under all noise and pressure conditions.

**Table 2:** Mean score of gustatory and olfactory function in all conditions.

	Normobar		Hyperbar	
*Objective* (Mean (SD))	Silence	Noise	Silence	Noise
	**Olfactory function**			
**Threshold score**	6.40 (1.56)	6.77 (1.12)	6.57 (1.47)	6.67 (1.93)
**Discrimination score**	12.69 (1.74)	13.19 (1.10)	13.06 (1.84)	12.50 (1.63)
**Identification score**	13.75 (1.24)	13.88 (1.02)	13.56 (1.03)	13.50 (1.32)
**TDI**	32.84 (3.17)	33.83 (2.01)	33.19 (3.08)	32.67 (2.88)
	**Gustatory function**			
**Gustatory score**	15.00 (2.31)	14.94 (1.69)	14.5 (2.16)	14.56 (2.25)
**Sweet**	3.50 (0.82)	3.56 (0.51)	3.50 (0.82)	3.50 (0.63)
**Sour**	2.75 (0.45)	2.75 (0.68)	2.50 (0.63)	2.56 (0.63)
**Salty**	3.31 (0.95)	3.50 (0.63)	3.31 (0.79)	3.25 (0.77)
**Bitter**	3.63 (0.62)	3.38 (0.81)	3.25 (1.00)	3.44 (0.81)

The mean odor identification score was not affected in hyperbaric or normobaric conditions (F(3) = 2.2, p = 0.53, n = 16). No differences could be found for odor discrimination (F(1,15) = 0.15, p = 0.7), the TDI (F(1,15) = 0.47, p = 0.5), or the threshold (F(1,15) = 0.02, p = 0.88) between different barometric conditions. Noise did not show a significant effect on the mean identification, TDI (F(1,15) = 0.17, p = 0.69), or discrimination scores (F(1,15) = 0.01, p = 0.92) under normobaric or hyperbaric conditions. The odor threshold score was not influenced by ambient noise in both barometric conditions (F(1,15) = 0.64, p = 0.44). No interaction was observed.

**Fig 2:**
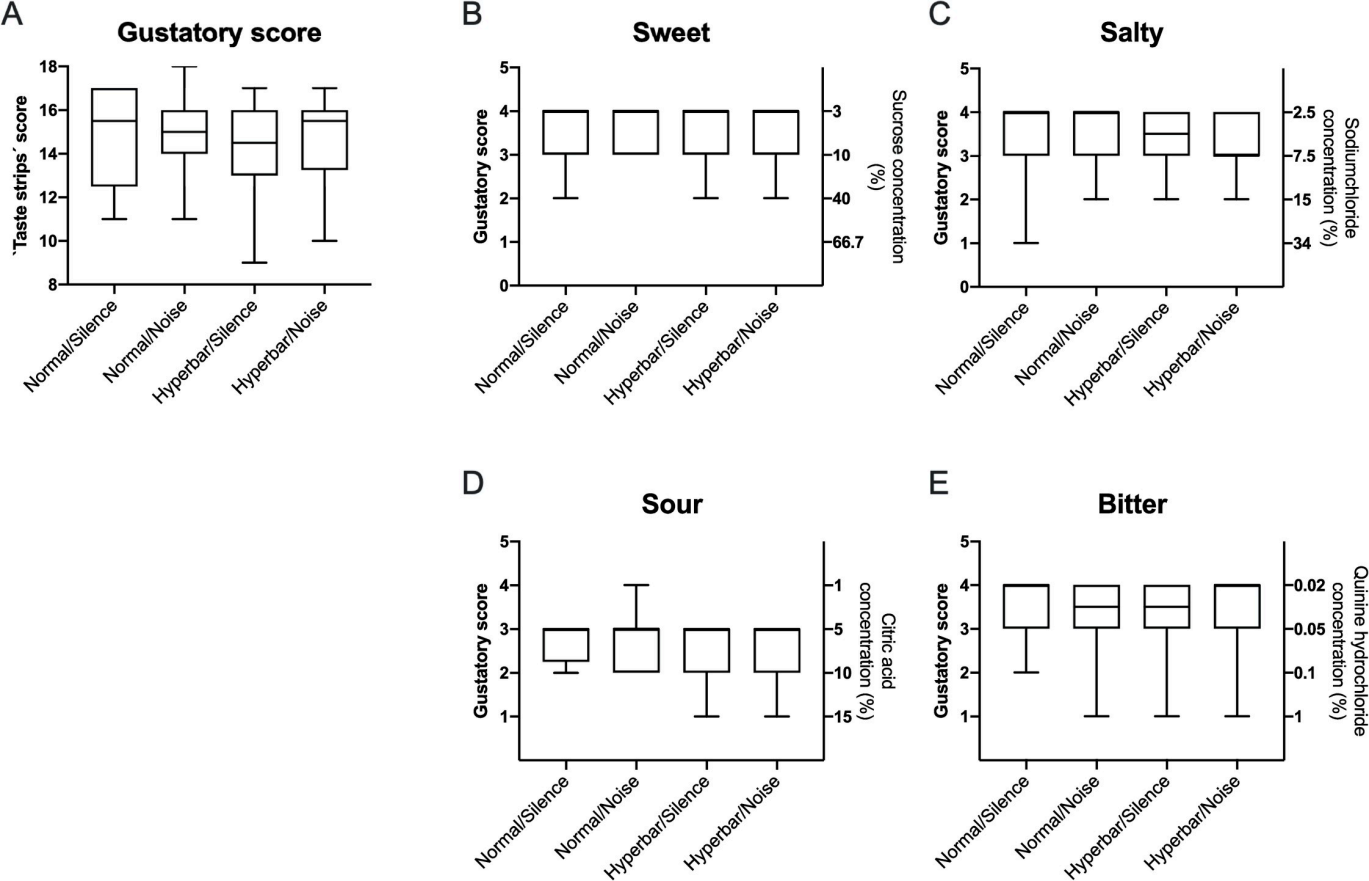
Gustatory score and underlying tastant thresholds for all participants. (A) Cumulative function score based on `Taste Strips’ identification. (B-E) The underlying thresholds for the different tastants as well as the corresponding tastant concentration are also shown. The whiskers show the mean and standard deviations.

Fig 2 shows the gustatory score and the underlying tastant scores for all participants.

Pressure and noise had no significant effect on the mean gustatory score (F(3) = 0.9, p = 0.83, N = 16). No interaction or other effects were found for ‘sweet’ (F(3) = 0.3, p = 0.96, n = 16), ‘sour’ (F(3) = 2.46, p = 0.48, n = 16), ‘salty’ (F(3) = 1.88, p = 0.59, n = 16) or ‘bitter’ (F(3) = 4.29, p = 0.23, n = 16) tastants.

## Discussion

This study did not show any significant effects of hyperbaric pressure (2 bars) on odor or taste.

### Olfaction

The experimental setup used here did not allow a confirmation of former findings, which showed that olfactory sensitivity increased under hyperbaric conditions [3, 8]. Previous studies explained the increase in olfactory threshold, based on two experimental gas laws [8]. First, according to Boyle’s law, pressure is inversely proportional to volume at a constant temperature. Therefore, when a gaseous volume containing a certain number of odor molecules is compressed, more odor molecules per gas volume could be available under hyperbaric conditions. Second, according to Henry’s law, the amount of dissolved gas in a liquid is proportional to its partial pressure above the liquid. The solubility of gaseous odor molecules in the tissues and percentage of gaseous odor molecules that bind the olfactory receptor neurons could increase with pressure, and thus, lead to increased threshold values.

The experimental hyperbaric environment in this study was set at 2 bar. Thus, the ambient pressure was increased by a factor of two, which, in turn would double the number of molecules inside the chamber. Consequently, the effect on olfaction might be expected to be much higher, based on the hyperbaric change. Since experiments started after compression in a fixed gas volume, the amount of odor molecules per volume would not be expected to increase, because the air distribution of particles would not differ significantly.

Potentially, according to Henry’s law, increasing the pressure could cause slower degassing of the dissolved odor molecules in the `Sniffin sticks´. But a faster rate of tissue uptake through odorant endocytosis on the one hand might be compensated by a slower rate of odorant release from the stick on the other hand. If the increased pressure inhibits degassing, then the release of molecules into the air would be slower, and this would be expected to reduce the concentration within the 2-cm distance from the nose.

A potential limitation of this study was the methodology for testing the olfactory threshold. First, the only odor tested (n-butanol) was not representative of daily life smell perception. Moreover, the repeated testing could have caused a learning effect that influenced the results. Our results showed that a high pressure reduced the identification score, but did not influence odor discrimination [8, 29]. Furthermore, our results indicated that noise did not have any significant effects on either olfactory or gustatory function. The odor threshold score was not affected by the white noise environment. Previous investigations found that functional magnetic imaging (fMRI)-generated noise had a significant negative impact on the olfactory detection threshold score. This effect might be caused by the high sound pressure level of loud fMRI-generated noise, which averaged 100 dB (peak 110 dB) [30]. This noise level was much higher than the 70 dB noise used in the present study. Additionally, different characteristics of different noise stimuli might influence odor perception [31]. An MRI generates acoustic patterns of multiple intensities and pitch, which can affect attention; therefore, an fMRI stimulus might exert undesired modulatory effects on odor processing in the brain [32–34]. In future studies, the hyperbaric ambient pressure could be set above 2 bar, to obtain more definitive evidence in support of these findings. This could be reached taking other gas mixures as (e.g. helium as inert gas) to avoid mental impairments by a nitrogen narcosis under higher pressure.

Another limitation of our experimental setup was the lack of acclimatization inside the hyperbaric environment. An adjustment and measurement as a function of time could provide more information with respect to the reduction of taste and smell sensitivities described by saturation divers [11]. These could be due to barometric changes of chemosensory physiology, the neurological effects like the high pressure nervous syndrome [35], or by fatique during long-term exposure [36].

In addition, more defined conditions might reduce potentially confounding factors, like humidity or stress.

The TDI score was reduced compared to the normative range, based on age and sex [22]. The subscore of odor threshold was also reduced, but the odor identification and discrimination scores were within the normative range. These different results might be caused by manufacturing differences of the odor-dispensing pens test battery or by variations in the methodology; e.g., holding the pen in different positions under the nostrils or allowing different time periods for smelling. We assumed that the observed threshold scores were reliable, due to the homogeneous measurements in the different conditions, the calibration between the two examiners, and the homogeneity observed in the mean scores of the volunteers.

It remains unclear if a more sensitive olfactory test could have picked up on more subtle deviations. Instead of the psychophysical tool applied in the present study, an electroencephalography (EEG) olfaction could have been utilized for more objective assessment, excluding potential effects of hyperbaric cognitive impairments on the reception of olfactants. However, using an olfactometer inside a pressurized chamber might cause pneumomatic errors [37]. It has to be also noted that statistically significant changes in psychophysical olfactory test scores do not necessarily reflect a clinically relevant change [38].

### Gustation

Previously, Woods et al. [39] found that auditory background noise affected gustatory perception, but other studies could not confirm that result [17]. Additionally, Woods et al. found a relationship between the preference ratings for the background noise and preference ratings for the food offered [39]. Therefore, we assumed that sound-induced emotions or a perceptional mismatch between sound and taste might underlie changes in taste. Confounders, like anxiety, claustrophobia, and uncomfortable positioning might also have influenced sensority performance. Another potential confounder might be reduced cognitive abilities caused by nitrogen narcosis. However, that effect should not have been relevant at 2 bar of pressure. The white noise used in our study was a rather flat spectrum sound, with no emotional valence; therefore, psychological confounders should not have affected either olfactory or gustatory function.

Our results showed that the hyperbaric atmosphere did not significantly affect gustatory function. However, pressure could have influenced the separate taste modalities, like ‘salty’. Rahne et al. [18] reported that hypobaric pressure significantly reduced the scores for ‘sour’ and ‘salty’ compared to the scores for ‘sweet’ and ‘bitter’. O’Reilly et al. [10] found an increase in ‘sweet’ sensitivity and a decline in ‘sour’ sensitivity after a 17-day dive. They also found a lower threshold for ‘bitter’ and a higher threshold for ‘salty’ stimuli at maximum pressure, but they could not exclude an influence from stress or other psychological effects. Halbreich et al. [9] used dissolved gustatory stimuli instead of taste strips and found a significant increase in recognition-errors for all four taste modalities with increasing hyperbaric pressure. The discrepancies between those findings and the findings of the present study might be explained by the different application methods.

Cross-modal effects can occur between auditory and taste perceptions [40], where the psychoacoustic influence of sound might be associated with a particular food at the cortical level. To avoid cross-modal effects, this study used artificial tastants (no food) and white noise.

The gustatory subscore distrubutions show a clear ceiling effect. The use of more sensitive gustatory tests might allow revealing smaller effects of ambient noise or hyperbaric atmosphere. However, small reductions of gustation may have very little to no clinical relevance. Due to the ceiling effects, the test utilized in this study for gustation did not allow detection of improvements.

## Conclusion

This study showed that gustatory and olfactory functions were not significantly affected by a hyperbaric atmosphere or by ambient noise. From a practical point of view, the influence of pressure in moderate hyperbaric occupations should be negligible.

## Supporting information

S1 Data Individual results for taste and smell tests. (XLSX)
